# Towards Online Multiresolution Community Detection in Large-Scale Networks

**DOI:** 10.1371/journal.pone.0023829

**Published:** 2011-08-24

**Authors:** Jianbin Huang, Heli Sun, Yaguang Liu, Qinbao Song, Tim Weninger

**Affiliations:** 1 School of Software, Xidian University, Xi'an, China; 2 Department of Computer Science and Technology, Xi'an Jiaotong University, Xi'an, China; 3 Department of Computer Science, University of Illinois at Urbana-Champaign, Urbana, Illinois, United States of America; University of Zaragoza, Spain

## Abstract

The investigation of community structure in networks has aroused great interest in multiple disciplines. One of the challenges is to find local communities from a starting vertex in a network without global information about the entire network. Many existing methods tend to be accurate depending on a priori assumptions of network properties and predefined parameters. In this paper, we introduce a new quality function of local community and present a fast local expansion algorithm for uncovering communities in large-scale networks. The proposed algorithm can detect multiresolution community from a source vertex or communities covering the whole network. Experimental results show that the proposed algorithm is efficient and well-behaved in both real-world and synthetic networks.

## Introduction

Nowadays, there are growing interests in analyzing the complex networks, such as the World Wide Web [1], citation networks [2], online social networks [3], and metabolic networks [4]. A network is always expressed as a graph consisting of vertices, representing entities, and edges, indicating relationships between entities. A common feature of these complex networks is community structure [5]–[7]. A network community is usually regarded as a group of vertices that are more densely connected within the group than the rest of the network. These communities usually indicate a meaningful topological relationship between entities within the network [5], [8]. Community discovery within networks is an important problem with many applications in a number of disciplines ranging from social network analysis to image segmentation and from analyzing protein interaction networks to the circuit layout problem.

A large number of methods have been presented for detecting communities in complex networks [9], [10]. For example, many community detection algorithms have been created utilizing greedy optimization of a modularity function 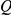 [11], [12]. However, any algorithm using 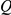 must necessarily be a global method, requiring complete knowledge of the entire network. Meanwhile, it has been shown that modularity 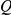 is not a scale-invariant measure, and hence, by relying on its maximization, detection of communities smaller than a certain size is impossible. It is well known as the resolution limit problem [13]. For many real-world networks, such as the Web graph and social networks, which are extremely huge and fast evolving, their global structures are almost impossible to consider. Therefore, existing global approaches have difficulty in handling these large, real-world networks.

Recently, researchers have proposed several methods for identifying communities based on the local link structure of a network [14]. P. Bagrow *et al.* [15] explored the local module of a source vertex through breadth first search. A local community is found until the expansion rate falls below some predefined threshold. This approach works well only if the source vertex is enclosed in the middle of the module. A. Clauset [16] proposed a new measure to detect communities by using a local optimization of a metric called *local modularity*, which only considers vertices within the boundary of a sub-graph. However, this method is sensitive to the join number parameter 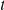, which must be specified by the user. The LWP algorithm proposed in [17] defines a different local modularity measure, which is closely related to the idea of a weak community [18]. X. Xu [19] proposed a density-based network clustering method for community detection. However, like other density-based clustering methods, it is sensitive to a minimum similarity threshold parameter, and it provides no automated way to find the parameter. Another important feature of complex networks is the intrinsic hierarchical community structure. So the revealed communities depend on the scale at which the network is examined [20]–[23]. A. Lancichinetti *et al.* [20] proposed a hierarchical and overlapping community detection method LFM based on a local fitness measure, which generates multiple communities to show hierarchies of the network by randomizing the starting vertices and varying a resolution parameter 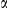.

To deal with the problems above, we present an approach for online multiresolution local community detection in large-scale networks. We introduce a similarity-based quality function of a community, called *tightness*, and present a algorithm LTE (Local Tightness Expansion), for revealing the natural community from a starting vertex or communities covering a network via local optimization of the tightness measure. Moreover, our method can identify overlapping communities in multiresolution by adjusting an optional resolution parameter.

The rest of the paper is organized as follows. First, the tightness measure and vertex adoption criterion for local communities are formalized. Then the algorithm LTE is described in detail. The experimental results are reported. Finally, the last section summarizes the conclusions and suggests future work.

## Methods

### Measurement of local community

**Figure 1:**
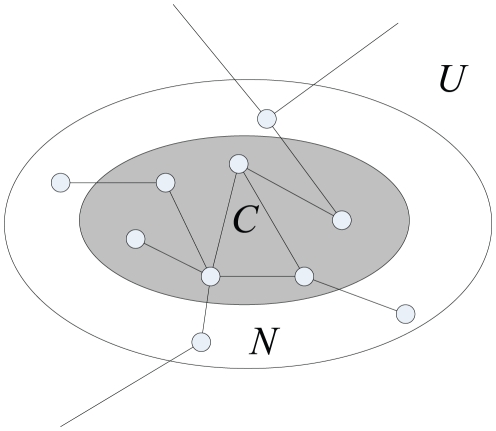
An illustration of the division of an abstract graph into the local community 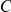, its neighborhood 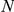, and the edges that connect 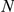 to the unknown vertices in 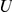.

Usually, a network can be represented by a graph 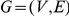, where 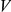 is the set of vertices and 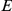 is the set of edges. Community detection is the task of finding densely connected sub-graphs in 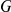. Our algorithm is based on the assumption that a community containing a given vertex 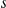 is a sub-graph that can be locally expanded from 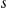 with a predefined criterion. In such local structures, a network is covered by distinct vertex groups. In the processing, we only deal with the vertices in a limited region, including the community 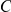, the neighborhood 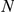, and the unknown part 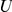. The structure is shown in Fig. 1.

Generally, a community is measured by a specific property of the vertices within it. For this task, different community measurements have been proposed [16], [20], [24]. Here, we introduce a structural similarity-based metric. First, we formalize some notions of the local community.

**Definition 1** *(Neighborhood) Let* 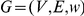 *be a weighted undirected network and* 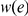 *be the weight of the edge* 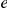 *. For a vertex* 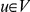 *, the structure neighborhood of vertex* 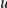 *is the set* 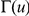 *containing* 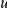 *and its adjacent vertices which are incident with a common edge with* 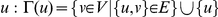 *.*

Then we can use the similarity functions based on the common neighborhood to measure the similarity of any pair of adjacent vertices. In this paper, we adopt a structural similarity measure from the cosine similarity function used in [21] which effectively denotes the local connectivity density of any two adjacent vertices in a weighted network.

**Definition 2** *(Structural Similarity) Given a weighted undirected network* 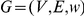 *, the structure similarity* 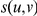 *between two adjacent vertices* 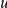 *and* 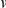 *is:*

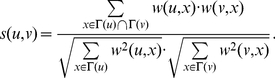
(1)
*When we consider an unweighted graph, the weight* 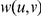 *of any edge* 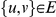 *can be set to 1 and the equation above can be transformed to*

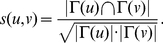
(2)
*It corresponds to the so-called edge-clustering coefficient introduced by Radicchi et al.* [18].

**Definition 3** *(Tightness) By employing the structural similarity, we introduce tightness, a new quality function of a local community* 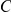 *, which is given as follows:*

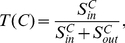
(3)
*where* 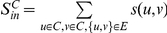 *is the internal similarity of the community* 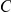 *which is equal to two times of the sum of similarities between any two adjacent vertices both inside the community* 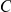 *;* 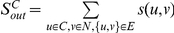 *is the external similarity of the community* 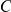 *which is equal to the sum of similarities between vertices inside the community* 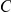 *and vertices out of it.*

The tightness measure is extended from the weak community definition proposed by F. Radicchi [18]. Similar to other community definitions [20], [25], the tightness value of a community 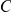, denoted by 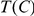, will increase when sub-graph 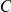 has high internal similarity and low external similarity. The whole network without outward edges will achieve the maximal value 1, but the problem here is to find the local optimization of the measurement for each community.

**Figure 2:**
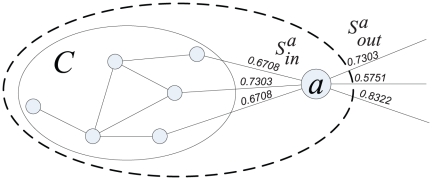
The tightness variant of a community 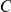 when a vertex 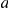 joins 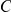.

Suppose a community 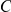 is detected from a certain vertex 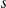. We explore the adjacent vertices in the neighborhood set 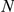 of 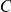, as shown in Fig. 2. So the variant tightness of the community 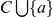 becomes
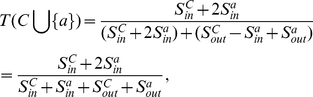
(4)where 
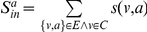
 and 
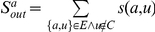
. Then the tightness increment of a vertex 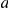 joining in 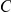 is
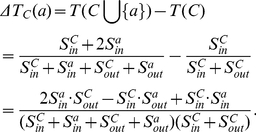
(5)If 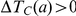 holds, then 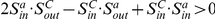 which is equivalent to 
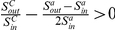
. So we define the tightness gain in the following.

**Definition 4** *(Tightness Gain) The tightness gain for the community* 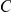 *adopting a neighbor vertex* 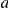 *can be denoted as*

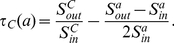
(6)
**

Let 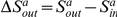 and 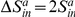, the equation above is equivalent to
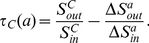
(7)It means that the ratio of external similarity to internal similarity of community 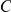 is greater than the ratio of external similarity increment to internal similarity increment caused by adopting vertex 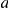. Obviously, this case will result in the increase of the tightness value of community 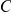. Therefore, 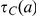 can be utilized as a criterion to determine whether the candidate vertex 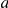 should be included in the community 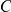 or not.

In the following, we introduce an optional resolution parameter 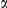 to control the scale at which we want to observe the communities in a network.

**Definition 5** *(Tunable Tightness Gain) The tunable tightness gain for the community* 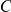 *merging a neighbor vertex* 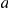 *can be denoted as*

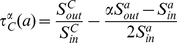
(8)
**

A parameter 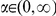 is introduced as the coefficient of 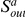 which can increase or decrease the proportion of the external similarity of the candidate vertex 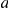. Here, the criterion for accepting a vertex 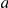 is changed to 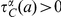. For 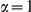, the criteria is moderate and can be used in most normal cases. However, if we set 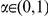, the value of 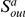 is reduced by this coefficient which increases the chance of a candidate vertex 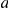 joining 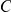 and bigger communities will be formed compared to the normal case with 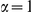. On the contrary, it will result in the formation of smaller communities in a network when we set 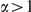. Therefore, large values of 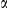 yield small communities, small values instead deliver large communities. In most cases, the whole network forms a single community containing all the vertices with 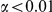. In contrast, several small groups containing two or more vertices are identified when we set 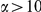.

### The Algorithm

Like other local community inferring methods [16], [17], [20], our local community detection algorithm is able to start from an arbitrary vertex 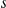 in a network 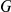. To find the densely connected local community containing vertex 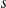, our algorithm works with two iterative steps: update step and join step. First, the starting vertex 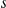 is added in 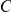. In the update step, we refresh the neighborhood set 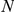, and calculate the structural similarities between vertices in the community 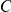 and their neighbor vertices in 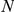. In the joining step, we try to absorb a vertex in 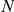 having highest structural similarity with vertices in 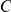 into the community 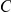. If 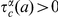, then the vertex 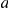 will be inserted into 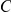. Otherwise, it will be removed from 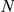 and other vertices will be considered in the descending order of the structural similarity. The two procedures above will be repeated in turn until set 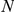 is empty. Then, the whole community 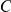 is discovered. Details of the local community detection algorithm are given in the following.

Step 1. Pick a vertex 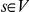 as the starting vertex. Let 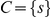 and 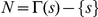.
Step 2. Select the vertex 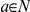 that possess the largest similarity with vertices in 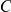.
Step 3. If 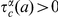, set 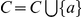 and 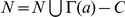.
Step 4. Repeat Step 2 and Step 3 until 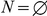.

Unlike methods proposed in [16], [17], [20], which calculate the quantitative metrics for every vertex in the neighbor sets and select the vertex who produces the greatest increment of the metric to join the community 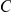, our method picks the neighbor vertex with the largest similarity as the candidate vertex and calculate 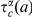 to determine whether it should be added to the community 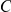 or not. The structural similarity reflects the local connectivity density of the graph. The larger the similarity between a vertex outside the community and a vertex inside it, the more common neighbors the two vertices share, and the more probability they are at the same community. Furthermore, unlike the quantitative metrics mentioned above, which need to be recalculated for each neighbors of a new vertex added in the community, the similarity of each pair of vertices and 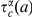 in our algorithm are both calculated only once by using a dynamical priority queue. So the execution of our algorithm is accelerated and the accuracy remains high.

The algorithm proposed above can be rerun from different vertices not yet belonging to any community, to form the communities covering the whole network. The process of our local tightness expansion algorithm LTE are given as follows.

Step 1. Pick a vertex 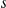 that has not been assigned to any community as the starting vertex.
Step 2. Detect the local community containing 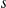 by using algorithm described above.
Step 3. Repeat from step 1 until all vertices in 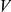 are visited.

Actually, if we allow a new community to be able to absorb vertices possessed by other communities, then it will form overlapping communities. Otherwise, non-overlapping communities are formed. In a word, our algorithm can identify either the local community from a specified vertex or communities covering the whole network. A problem for our non-overlapping community detection method is that the result, to a certain extent, depends on the visiting order of vertices [11], [16], which means that different starting vertices sequence may lead to different partition of the network. However, our experimental results show that the effect is slight, especially for networks with clear community structures.

The running time of our community detection algorithm LTE is mainly consumed in selecting the neighbor vertex with the largest similarity in the process of forming the local communities. We perform this function with a dynamic priority queue which is implemented with a Fibonacci heap. So the computational complexity for our non-overlapping local expansion method is almost the same as generating the minimal spanning tree of a graph with the Fibonacci heap, which has a computational complexity of 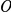
(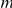
+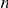
log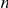), where 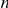 is the number of vertices inferred, and 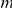 is the number of edges in the network. For scale free network, the running time complexity is 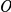
(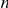
log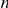).

When the overlapping communities are considered, the running time is longer than the non-overlapping process because some vertices will be visited multiple times. However, the computational complexity is hard to estimate as it depends on the resolution parameter 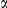 and intrinsic link structure of the network.

**Figure 3:**
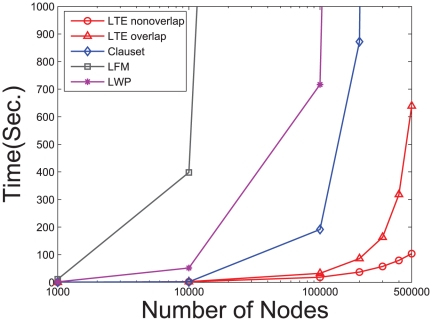
Running time comparison for our algorithm LTE with other state-of-the-art local community detection algorithms.

To illustrate the running time of the proposed algorithm LTE and to compare it with other algorithms, we generate seven networks where the numbers of vertices 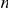 range from 1,000 to 500,000 and the numbers of edges 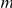 are always ten times 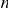. The running times of our algorithm on the synthetic networks compared with other local community detection methods (e.g., Clauset's method, LFM and LWP) are plotted in Fig. 3. It shows that our algorithm LTE with 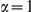 for detecting non-overlapping communities can process a network with 500,000 vertices within two minutes. We can observe that both the overlapping and non-overlapping version of our algorithm LTE run much faster than LFM , LWP and Clauset's method.

## Results

In this section,we evaluate our method using some real-world datasets and computer-generated benchmark datasets. Our algorithm is implemented in ANSI C++. All the experiments were conducted on a PC with a 2.4 GHz Pentium IV processor and 2 GB of RAM.

### Test On Real-World Networks

To evaluate the accuracy of our algorithm, we first conduct experiments on three popular real-world networks.

#### Zarchary's karate club

**Figure 4:**
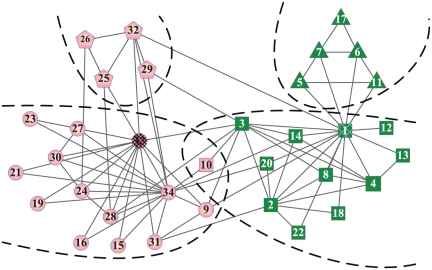
The Zachary's karate network and four communities discovered by our algorithm on it.

Zarchary's karate club is a well known social network that describes the friendship relations between 34 members of a karate club observed over two years by Zarchary [26], as shown in Fig. 4. In that period, the club members split into two distinct groups due to a contrast between the owner (vertex 1) and the instructor (vertex 33) of the club.

We start from vertices 1 and 33 respectively to detect the overlapping communities, and then detect communities from other vertices which have not been absorbed in communities. When we set 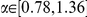, four communities are discovered in this network by our algorithm, as shown in Fig. 4. It is noteworthy that vertex 10 is shared by two communities. It is absorbed by these two communities since it connects the two communities in the same way and the positive tightness gains are harvested when it is merged by both of these two communities.

When we set the parameter 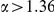, the number of detected community also increases along with the increase of the parameter. Finally, we get a stable result in which all communities are made up of two or a little more vertices. This is because the tightness gain of the first absorbed vertex starting from an arbitrary vertex must be positive, no matter how large the parameter 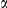 is. If there are neighbors only having links to the above two vertices, they are also enclosed in this community. We find that in any networks, when the value of parameter 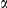 is large enough, our algorithm always finds covers of networks as that described above. When we set 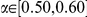, our algorithm detects two communities in which vertices 5, 6, 7, 11, 17 form a community and the remaining vertices are enclosed in the other community. When 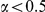, the whole network is identified as a single community.

The algorithm LFM detects the same four communities in this network with parameter 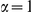. The Clauset's method also finds four communities in this network. The only difference is that three vertices 3, 9, 10 are identified as shared overlapping vertices by the owner's community and instructor's community. Though the result of our algorithm on Karate network does not match the ground truth of the dataset, it is similar to results often found by other methods, which means that it is topologically meaningful.

#### NCAA college-football network

**Figure 5:**
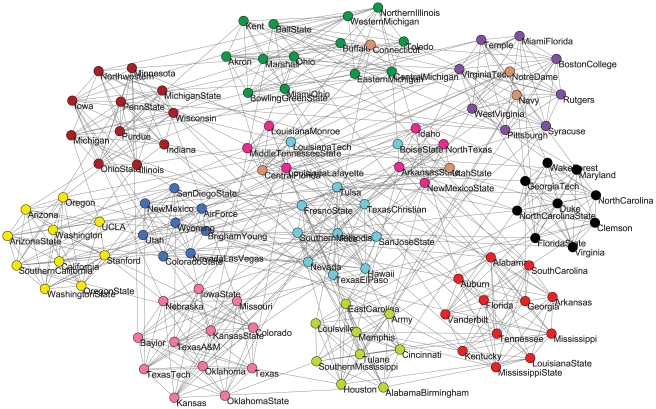
The NCAA college-football network and thirteen communities obtained by our algorithm on it with 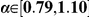. The conferences are represented by the vertex groups and the detected communities are distinguished by different colors.

The NCAA college-football network is a representation of the schedule of American Division I college football games for the 2000 season [1]. As shown in Fig. 5, 115 vertices in the network represent teams (identified by their college names), which are divided into eleven communities (or conferences) and five independent teams (Utah State, Navy, Notre Dame, Connecticut and Central Florida). 616 edges represent regular season games between the two teams they connect. The question is to automatically recover the conferences within the network.

First, we use our algorithm LTE to detect non-overlapping communities in this network. The visiting sequence of the vertices is in the ascending order of the vertex number in the dataset. When we set 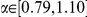, our algorithm identifies thirteen communities within this network, as shown in Fig. 5. Among them, seven conferences (e.g., Atlantic Coast, Mountain West, Big 10, Big 12, Pac 10, SEC and Conference USA) are correctly identified. Interestingly, the Mid-American conference is divided into two small divisions in real-world: MAC East and MAC West, which are correctly identified as communities respectively by our algorithm. The remaining two communities closely resemble the Sunbelt and Western Athletic conferences. Five independent teams that do not belong to any conference tend to be grouped with the conference with which they are most closely associated. There are also a few failure cases for our algorithm. For example, the team Boise State in the Western Athletic conference is incorrectly merged into the Sunbelt conference. We found that Boise State played more games against Sunbelt teams than teams in its own conference, which likely explains the inaccuracy. In general, our algorithm is inaccurate in cases like this where the network structure genuinely does not correspond to the real-world organization of conferences. In other respects, our algorithm performs remarkably well.

When we increase the value of 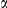 from 1.10 gradually, the number of detected community increases and the size of each community decreases correspondingly. Finally, we get the same stable result described above. When we set 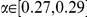 and 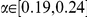, our algorithm can detect six and two communities respectively in this network. The whole network forms a single community when 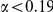.

Based on the intrinsic conferences in the football network, we compare the accuracy of our algorithm with other local methods by calculating precision and recall scores for each conference [27]. We take every vertex in the network as the starting vertex, and execute the algorithm LTE with 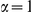 to infer the local community. Finally, the overall precision and recall scores of a conference is the average of the detected communities starting from each vertex in the conference. As shown in Table 1, our algorithm LTE achieve much higher accuracy than the Clauset's method and LWP method in every conference. The result of algorithm LFM is comparable to that of our method. Both LTE algorithm and LFM algorithm correctly detect the conferences Atlantic Coast, Big East, Mountain West, Pac 10, and SEC in this network. In conferences Big 10, USA, Mid-American and Western Athletic, the precision and recall values of LFM are a little higher than that of our method. But our algorithm LTE obtains better result on conferences Big 12 and Subelt. The LFM algorithm performs well in this network because it using a dynamic process of vertex selection for detecting a local community. When a new vertex is absorbed in a community, the fitness variant of all the vertices in the community will be recalculated and the vertices having negative fitness contribution will be get out of the community which will result in more densely connected communities in sparse real-world networks.

**Table 1:** Accuracy comparison of local community detection methods for each conference in NCAA college-football network.

NCAA college-football network		Clauset		LWP		LFM		LTE	
conference name	size	Precision	Recall	Precision	Recall	Precision	Recall	Precision	Recall
Atlantic Coast	9	0.7037	0.7054	**1**	**1**	**1**	**1**	**1**	**1**
Big 10	12	0.3889	0.39	0.8472	0.9226	**0.9167**	**1**	0.8819	0.9881
Big 12	12	0.9236	0.8251	**1**	**1**	**1**	0.8571	**1**	**1**
Big East	8	0.5625	0.4895	0.1719	0.1033	**1**	**1**	**1**	**1**
Conference USA	10	0.67	0.6686	0.1444	0.119	**0.9**	**1**	0.89	0.9889
Mid-American	12	0.9236	0.753	1	0.8571	**1**	**0.8571**	0.5	**0.8571**
Mountain West	8	0.8906	0.8026	0.1563	0.1169	**1**	**1**	**1**	**1**
Pac 10	10	0.82	0.7322	**1**	**1**	**1**	**1**	**1**	**1**
SEC	12	0.9236	0.7874	**1**	**1**	**1**	**1**	**1**	**1**
Sunbelt	7	0.5306	0.5365	0.1429	0.0897	0.4286	0.5	**0.5102**	**0.6381**
Western Athletic	10	0.62	0.5874	0.6091	0.6717	**0.8**	**0.8889**	0.74	0.8378

#### Amazon co-purchase network

We also use our method to study a large-scale co-purchase network from Amazon.com. This network was collected in January of 2006, and has been studied in [16], [17]. In this network there are 585,283 vertices and 4,566,749 edges with an average degree of 7.80. The vertices represent items such as books, CDs and DVDs sold on Amazon's Web site and the edges connect items that are frequently purchased together by customers.

As illustrative examples, we choose five digital media (four CDs and one DVD) and five books as source vertices, as shown in Table 2. These items were adopted in [17]. The CD “Alegria” and the books “Small Worlds” and “Harry Potter” were used in [16].

**Table 2:** Source vertices and sizes for illustrative examples of detected communities using our algorithm LTE in Amazon co-purchase network.

source vertex	vertex number	type	degree	community size
Cirque Reinvente by Cirque du Soleil	B00000F3V8	DVD	15	**18**
Alegria by Cirque du Soleil	B000003FRF	CD	11	**25**
Love Wants to Dance by Maria Muldaur	B0002M5TB6	CD	14	**20**
Toddlers Sing by Music for Little People	B00000C41T	CD	10	**6**
Preschool Songs by Cedarmont Kids	B000008UPJ	CD	17	**23**
Molecular Biology of Cell by Bruce Alberts	0815332181	book	222	**47**
An Introduction to Bioinformatics Algorithms by Neil C. Jones et al.	0262101068	book	89	**43**
Molecular Biology of the Gene, Fifth Edition by James D. Watson	080534635X	book	24	**9**
Small Worlds by Duncan Watts	0691117047	book	15	**27**
Harry Potter and the Order of the Phoenix by J. K. Rowling	0439358078	book	34	**37**

**Figure 6:**
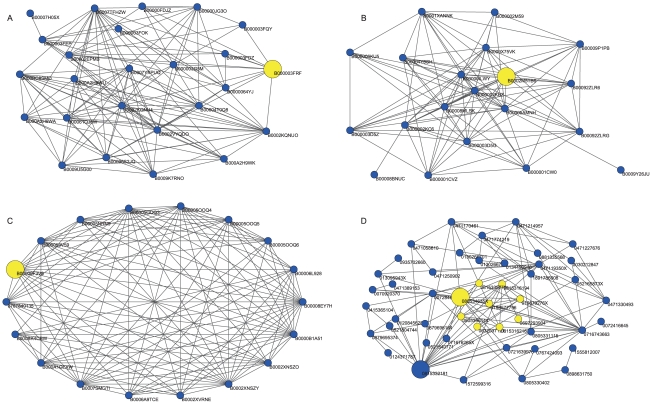
Identified local communities from different starting vertex in the Amazon co-purchase network. (A) Local community for the compact disc Algeria (ID B000003FRF), (B) local community for the DVD Cirque Reinvente (ID B00000F3V8), (C) local community for the compact disc Love Wants to Dance (ID B0002M5TB6), and (D) local community for the book Molecular Biology of Cell (ID 0815332181) and the book Molecular Biology of the Gene (ID 080534635X)(marked in yellow).

We find a community from each of the starting vertices above. On the whole, the vertices in the communities are mostly works by the same singer or author. Due to space limitations, we present only five identified communities below. Fig. 6(A) shows the local community for the CD “Alegria”. All 25 items found in this community are compact disks by Cirque du Soleil, the same as the results found in [17]. Fig. 6(B) shows the local community for the DVD “Cirque Reinvente”. Compared with the result found by in [17], the community found by our method is more accurate because it contains all 18 DVDs produced by Cirque du Soleil and excludes the books by Cirque du Soleil. Fig. 6(C) shows the local community for the compact disk “Love Wants to Dance”. All 20 items are compact disks of songs by Maria Muldaur, which covers the items found in [17]. The compact disk “Sweet Lovin Ol Soul” by Maria Muldaur is only identified by our method. Actually, it should be included in the community because it has more links with the vertices in the community than with the vertices outside the community.

Note that unlike the Clauset's algorithm proposed in [16], which may identify multiple communities as explores the network, our algorithm only identifies one community for each source vertex with a certain parameter. For example, starting from the same vertex: the compact disc “Alegria” by Cirque du Soleil, the Clauset's algorithm will identify multiple communities including a community of compact discs by Cirque du Soleil that corresponds to our result, a community of DVD by Cirque du Soleil that corresponds to the community we detect using the DVD “Cirque Reinvente” by Cirque du Soleil as the source vertex, and another community containing books and movies by Cirque du Soleil.

It is also discovered that some communities that start from certain vertices will be totally included in or share some vertices with other communities starting from other vertices. As is shown in Fig. 6(D), the community starting from the vertex “Molecular Biology of the Gene” (ID 080534635X) is wholly enclosed in the community starting from the vertex “Molecular Biology of Cell” (ID 0815332181).

Therefore, the local communities identified by our local expansion algorithm LTE are more uniform and reasonable, and will therefore provide more accurate information for future recommendation.

### Test On Synthetic Networks

So far, we have presented the experimental results of our algorithm on several real-world networks. In this section, we use some synthetic datasets to evaluate the performance of our algorithm.

#### LFR benchmark graphs

We use the Lancichinetti-Fortunato-Radicchi (LFR) benchmark graphs [24], [28] to evaluate the accuracy of our algorithm. Two kinds of networks are generated with different ranges of community size, where S means that the sizes of the communities in the dataset are relatively small and B means that the sizes of the communities are relatively large. The detailed parameters of the benchmark networks are given in Table 3.

**Table 3:** The detailed parameters of the generated benchmark networks.

Network	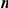	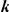	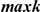	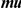	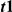	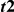	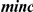	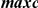	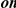	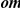
10000S	10000	20	50	0.1 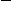 0.8	2	1	10	50	0 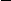 0.3	2
10000B	10000	20	50	0.1 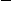 0.8	2	1	20	100	0 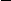 0.3	2
100000S	100000	40	100	0.1 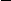 0.8	2	1	50	100	0 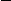 0.3	2
100000B	100000	40	100	0.1 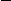 0.8	2	1	100	200	0 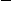 0.3	2

Some important parameters of the benchmark networks are: 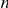: number of vertices; 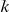: average degree of the vertices; 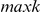: maximum degree; 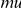: mixing parameter, i.e., each vertex shares a fraction of its edges with vertices in other communities (the higher the mixing parameter of a network is, the more difficult it is to reveal the community structure); 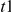: minus exponent for the degree sequence; 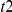: minus exponent for the community size distribution; 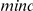: minimum for the community size; 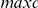: maximum for the community sizes; 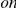: percent of the overlapping vertices; 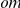: number of memberships of the overlapping vertices.

First, we set the overlapping parameter 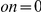, and generate eight networks for each type of dataset with mixing parameter 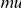 ranging from 0.1 to 0.8. When 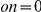, communities in the generated networks are non-overlapped. We adopt the *normalized mutual information*(NMI) to evaluate the quality of detected communities which is currently widely used in measuring the performance of graph clustering algorithms [24]. The accuracy of our method is compared with other state-of-the-art community detection methods: Clauset's local modularity [16], LWP [17], LFM [20] and BGLL [19]. For dealing with the possible effects of the vertex visiting order, we execute our method ten times with randomized vertex sequences and take the average as the final result. We select the result with maximum NMI for the LFM method which tries the parameter 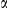 by itself and shows hierarchical module structure of the whole network.

**Figure 7:**
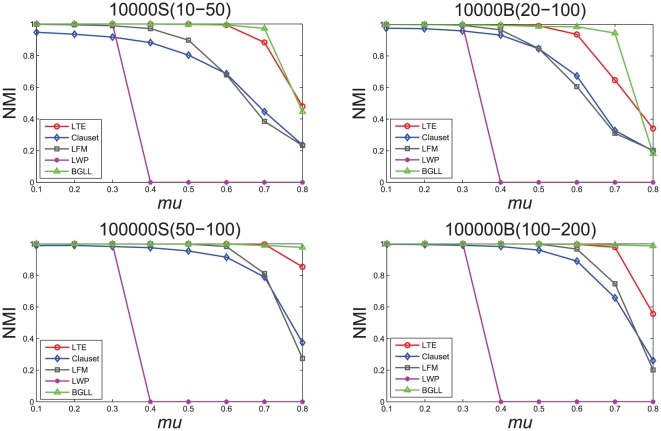
Test of the accuracy of different community detection algorithms on LFR benchmark networks.

The NMI scores of the four methods are plotted in Fig. 7. Our algorithm gets NMI = 1 when 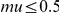 on the two S-networks and gets NMI = 1 when 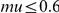 on the two B-networks, which means a perfect match with the original network structure. We can see that the accuracy of our method is higher than other three local community detection algorithms (e.g., Clauset's method, LFM and LWP) on the generated networks, because our method use a similarity-based quality function of local community and a proper vertex adoption criterion. Note that the NMI values of the results obtained by algorithm LWP on networks with 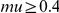 are all 0. This is because the community definition of the algorithm LWP is that the number of edges within the community should be more than the number of edges between vertices in the community and vertices outside it. However, none of the subgraphs can satisfy this definition in the network with 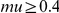. We can also observe that the obtained NMI values of our method LTE on the networks with 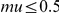 are almost the same as that of the method BGLL. But the accuracy of our algorithm is lower than the method BGLL on the networks with 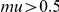 which shows that our algorithm has difficulty to deal with the network with indistinct community structure. BGLL is actually a global method by optimizing the modularity function locally and is known as an efficient and effective community detection method [13].

**Figure 8:**
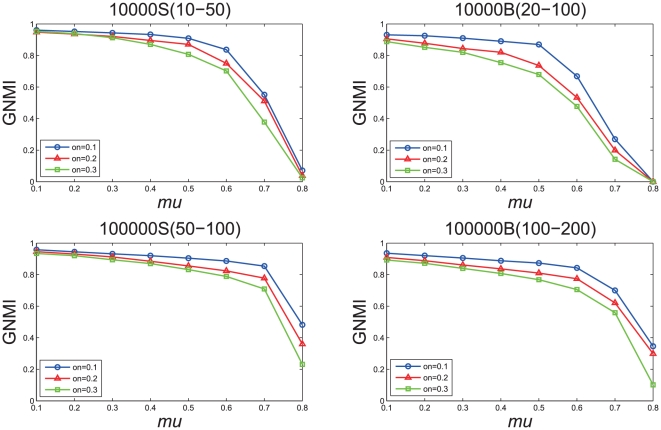
Test of the accuracy of our algorithm on LFR overlapping benchmark networks.

We also test the accuracy of our method with 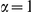 in detecting overlapping communities in the networks with parameter 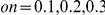, respectively. In this experiment, we adopt *Generalized Normalized Mutual Information* (GNMI) proposed in [20] to evaluate the quality of detected overlapping communities. Fig. 8 shows the evaluation results on the benchmark overlapping graphs. We can see that our algorithm gets high precision. However, the GNMI values decrease along with the increase of the parameter 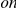, because there are more and more vertices belonging to multiple communities in the network when the value of parameter 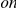 increases, which is increasingly difficult to be correctly identified.

#### Hierarchical benchmark networks

**Figure 9:**
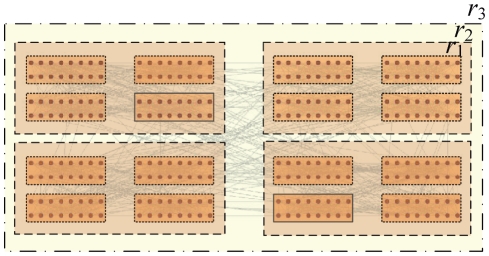
Schematic networks with hierarchical structures. Each of the four large clusters is made out of 64 vertices and has an internal subdivision in four clusters with 16 vertices.

Here, we also test our method on synthetic networks with built-in hierarchical community structure. A benchmark network adopted by A. Arenes *et al.* [29] is used. This benchmark network is a hierarchical version of the classical benchmark proposed by Girvan and Newman [5]. There are 256 vertices in the network, split into 16 groups with 16 vertices on the first level. The 16 groups are ordered into four super-groups at the second level, as shown in Fig. 9. The internal degree of vertices at first level 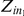 and the internal degree of vertices at second level 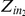 keep an average degree 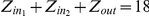. The networks are indicated as H 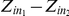, and we test the performance of our method on the H13-4 and H15-2 networks respectively. Our algorithm detects sixteen communities in both networks when we set 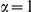, which matches exactly with the original network's structure. When we decrease the value of parameter 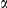, our algorithm can detect four communities and at last one community in the two networks, respectively. The parameters 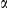 for three different resolutions 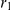, 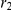 and 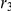 are given in Table 4.

**Table 4:** The detailed parameters 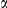 for three different resolutions 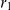, 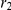, and 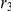 of the communities detected in the benchmark hierarchical networks H13-4 and H15-2, where 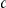 is the number of discovered communities.

Network	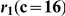	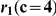	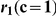
H13-4	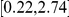	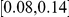	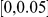
H15-2	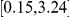	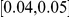	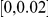

#### Random networks

For evaluating the performance of our method on random networks, we first test on the Erdos-Renyi random graph. Graphs with 100, 200, 500, 1000 vertices are generated where the connectivity probability 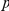 between each pairs of vertices ranges from 0.01 to 0.8. We run our algorithm with 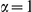 for detecting overlapping communities. We find that our method discover only one community in the random networks with parameter 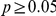. In the networks with 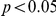, our method always finds covers of the whole networks consisting of a big community as well as several small ones. We also test our method on random scale-free graphs generated by the configuration model (http://snap.stanford.edu/). After testing our algorithm on several networks with different degree sequences, we find that our method always identifies a big community containing over 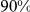 of the vertices along with several small communities in these networks. The above results show that our algorithm tends to find a big community as well as many small communities in the sparse random graph, while only one community can be detected in the random graphs with dense connections.

## Discussion

In this paper, we present a novel local community quality criterion, called similarity-based tightness, and design a greedy algorithm LTE to infer the local communities in large-scale undirected networks. The proposed method is able to identify communities both overlapping and non-overlapping, and we show that it is fast and scalable in large-scale networks. Moreover, we describe that our algorithm is a multiresolution solution that can be used freely to acquire communities at any resolution. Experimental results on the real-world and synthetic datasets show that our algorithm achieves good performance. In the future, we believe it is beneficial to apply this method when analyzing large-scale online networks like mobile communication networks and/or Web graphs.
